# Lymph Node Colonization Dynamics after Oral *Salmonella* Typhimurium Infection in Mice

**DOI:** 10.1371/journal.ppat.1003532

**Published:** 2013-09-19

**Authors:** Patrick Kaiser, Emma Slack, Andrew J. Grant, Wolf-Dietrich Hardt, Roland R. Regoes

**Affiliations:** 1 Institute of Microbiology, ETH Zurich, Zurich, Switzerland; 2 Department of Veterinary Medicine, University of Cambridge, Cambridge, United Kingdom; 3 Institute of Integrative Biology, ETH Zurich, Zurich, Switzerland; Emory University, United States of America

## Abstract

An understanding of how pathogens colonize their hosts is crucial for the rational design of vaccines or therapy. While the molecular factors facilitating the invasion and systemic infection by pathogens are a central focus of research in microbiology, the population biological aspects of colonization are still poorly understood. Here, we investigated the early colonization dynamics of *Salmonella* enterica subspecies 1 serovar Typhimurium (*S.* Tm) in the streptomycin mouse model for diarrhea. We focused on the first step on the way to systemic infection — the colonization of the cecal lymph node (cLN) from the gut — and studied roles of inflammation, dendritic cells and innate immune effectors in the colonization process. To this end, we inoculated mice with mixtures of seven wild type isogenic tagged strains (WITS) of *S.* Tm. The experimental data were analyzed with a newly developed mathematical model describing the stochastic immigration, replication and clearance of bacteria in the cLN. We estimated that in the beginning of infection only 300 bacterial cells arrive in the cLN per day. We further found that inflammation decreases the net replication rate in the cLN by 23%. In 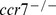 mice, in which dendritic cell movement is impaired, the bacterial migration rate was reduced 10-fold. In contrast, 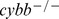 mice that cannot generate toxic reactive oxygen species displayed a 4-fold higher migration rate from gut to cLN than wild type mice. Thus, combining infections with mixed inocula of barcoded strains and mathematical analysis represents a powerful method for disentangling immigration into the cLN from replication in this compartment. The estimated parameters provide an important baseline to assess and predict the efficacy of interventions.

## Author Summary

Like humans, pathogens have a demography. Within their hosts, they migrate, replicate, and die. Understanding these processes quantitatively can help designing vaccines and treatment by identifying vulnerabilities of the pathogen population. For most pathogens, however, quantitative information on how they replicate and spread in their hosts is lacking. Here, we investigate the early colonization of hosts by *Salmonella* bacteria after oral infection in a mouse model for complicated *Salmonella* diarrhea. To estimate migration and replication rates, we infected the mice with mixtures of identical, but distinguishable strains of the bacterium and analyzed the results with a mathematical model that describes the demography of the bacterial population. Random loss of some of these strains during colonization of the mice allows us to estimate migration rates between anatomical compartments. We find that approximately 300 bacteria migrate per day from the gut to the cecal lymph node – the first step towards systemic infection. We further investigate how bacterial migration and replication is affected by inflammation and various agents of the immune system. Our study provides unprecedented information on the colonization dynamics of this bacterial infection and introduces a framework for further improving therapy and vaccination.

## Introduction

Understanding the population biological aspects of how a pathogen colonizes its host is crucial for prevention of infection. Only if we know where a pathogen enters, which anatomical compartments it colonizes, where and how fast it replicates, migrates, and gets killed, will we be able to optimally design interventions that block this process. Just as there are molecular Achilles' heels of pathogens, population dynamical parameters exist that characterize vulnerabilities of the infection process. Combining the knowledge of molecular mechanisms with an understanding of the population dynamics of an infection thus holds great promise for the design of vaccines and therapy. For most pathogens, however, the population biological aspects of infection are not well understood.

The population dynamics of pathogens within the host has been investigated most extensively for viruses, in particular for Human and Simian Immunodeficiency Viruses. We know how this virus enters [1], diversifies [2], [3], and is controlled by the immune response early in infection [4]–[8]. Less is known about the anatomical aspects of early colonization of humans because this requires frequent sampling of various compartments. In animal models, population dynamic parameters characterizing the anatomical spread of the virus are starting to be determined [9]. Similar insights have been obtained for influenza virus [10] and Hepatitis C Virus [11].

The study of the colonization dynamics of bacterial pathogens has an impressive early history. Meynell & Stocker used pairs or triplets of differentially marked strains of *Salmonella* to investigate if bacterial cells initiate an infection independently of each other [12], [13]. Later Moxon & Murphy extended this approach to *Haemophilus* influenzae [14]. By sampling bacteria from different compartments, they obtained qualitative information on the colonization pathways of the pathogen.

Today we have much better tools to monitor bacterial spread and replication at our disposal, and advanced population dynamical methods are being used to understand how infections unfold within their hosts [15]. However, these tools and methods have not been extensively used to analyze bacterial colonization dynamics. This is surprising because of the long history of this question, and the importance for these processes for vaccination and treatment.

Nevertheless, there are a few notable studies on the colonization of host by bacterial pathogens. Recently, Margolis & Levin [16] presented an extended study of the system used by Moxon & Murphy. In particular, they investigated if colonization relied on bacterial evolution. Furthermore, the colonization pathways of *Yersinia* pseudotuberculosis has been investigated qualitatively [17]. The arguments in this study are reminiscent of Moxon & Murphy's early study on the colonization dynamics of *H.* influenzae. Lastly, Grant et al. presented a quantitative study on the dynamics of *Salmonella* infection following intravenous inoculation of mice [18].

In the present study, we define the early population dynamics in a mouse model for *Salmonella* diarrhea [19], [20]. After inoculation of the mice, the bacterial population grows to a population size of about one billion cells in the gut. From the gut, bacteria colonize the cecal lymph node (cLN), and subsequently spread throughout the body of the mice from there. As the migration from the gut to the cLN initiates systemic infection, we focus on this step in the present study.

We were primarily interested in the rate at which the bacteria migrate from the gut to the cLN, and in the growth rate of the bacterial population in that compartment. To estimate these key population biological parameters we used inocula containing multiple wild type isogenic tagged strains (WITS) that have been used to elucidate population biological characteristics of bacterial pathogens in other systems [17], [18]. The mixed inocula revealed the stochasticity in the colonization process, which we could exploit for parameter estimation. To determine the population biological significance of inflammation and dendritic cells, we conducted experiments with mutant strains of *S.* Tm with well characterized virulence defects and different mice strains with known deficiencies in pathogen handling.

## Results

### Rationale and approach

Determining the migration, replication and clearance rates of bacteria *in vivo* constitutes a challenge. While the bacterial population size can be measured in many anatomical compartments, this measure is a complex composite of migration, replication and clearance, and hence does not allow quantifying the rates at which these processes occur. Therefore, we have developed a novel approach combining experimental infection with mixed inocula and mathematical modeling to determine bacterial migration to and replication with-in the cLN.

The infection experiments were performed using a mixture of seven *S.* Tm strains, each carrying a unique, 40 base pair long tag in the chromosome [18]. These strains are phenotypically identical to each other and to wild type *S.* Tm. The rationale for using mixed inocula was to reveal the stochasticity of the colonization process: for an appropriate mixing ratio, only some of the strains in the mix arrive in the cLN. The stochastic loss of WITS on their way to the cLN enables us to estimate the rate at which they immigrate. The strains that successfully enter the cLN subsequently increase in numbers. From these numbers, we can infer the growth rate of the bacterial population in the cLN.

**Figure 1:**
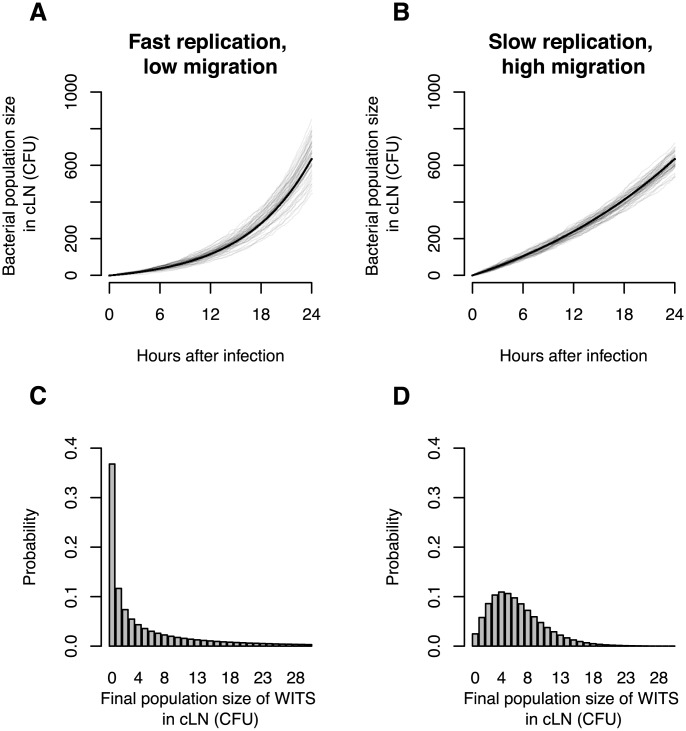
*In silico* analysis demonstrates the value of infection experiments with mixed inocula. **A**. Total bacterial population size in cLN for 50 simulations of a scenario with low migration rate into the cLN (100 bacterial cells per day) and fast net replication therein (doubling time of 5.5 hours). **B**. Total bacterial population size in cLN for the reverse scenario (immigration of 370 bacterial cells per day into the cLN and a doubling time of 16.6 hours). Gray lines indicate the output of individual simulation runs, and the black line is the analytically predicted mean bacterial population size. **C**. and **D**. The probability that WITS attain a specific population size in the cLN 24 hours after infection in the two scenarios. The frequency of a single WITS in the inoculum is assumed to be 1%. While the time courses of the bacterial population size at the top display only subtle differences, the probability distributions on the bottom are clearly distinct for the two scenarios. Thus, using mixed inocula one can disentangle division and immigration much more sensitively than by determination of the total bacterial population size only.

Before we present our findings, let us elaborate the advantages of using mixed inocula for studying of the colonization dynamics of the cLN. As we stated above, if we simply measure the bacterial population size in the cLN it is difficult to disentangle immigration from replication. Figure 1 illustrates this point. It shows simulations (based on the mathematical model described below) for a scenario with high immigration and low replication, and an alternative scenario with low immigration and high replication. The parameters are chosen such that the bacterial population sizes in the cLN are approximately equal for both scenarios one day after inoculation. Because the time courses of the bacterial population size in the cLN do not differ dramatically across the two scenarios it is, on the basis of such data, very challenging to determine which of them applies.

In these simulations, the variation of the bacterial population size arises only from the intrinsic stochasticity of bacterial division, clearance (i.e. the bacterial elimination or migration to other organs) and immigration from the gut. In real mice, the distinction between the two scenarios would be blurred even more because there would be larger variation in the time courses of the bacterial population size due to biological differences between mice and experimental sources of error. As a consequence, it is not feasible to distinguish the two scenarios on the basis of measurements of the bacterial population size alone.

With mixed inocula, on the other hand, the distinction between the two scenarios is clear-cut. Assume we inoculate with a mix containing seven WITS at a frequency of 1% and 93% untagged bacteria. In the scenario with low immigration, an individual WITS will fail to colonize the cLN in 37% of the cases. Thus, on average more than half of the WITS will be recovered from the cLN one day after inoculation. If immigration is high, however, WITS will fail to colonize in only 2.5% of the cases, i.e. all WITS will be recovered from the cLN in most animals. Using mixed inocula is therefore a much more sensitive approach to determining immigration and growth rates.

The mathematical model we employed to analyze these infection data is a stochastic birth-death process extended by immigration (see Materials & Methods). It predicts the bacterial population size of each WITS in the cLN as a function of the rate of immigration into the cLN, 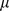, the rate of replication within the cLN, 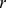, and rate of clearance in the cLN, 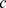. Thus, our model goes beyond simply considering the presence or absence of WITS in the cLN. While the absence of a WITS is equivalent to a population size of 0 CFU, a WITS that is present in the cLN can have any positive population size (1,2,3, …CFU).

From measurements of the population size of differently tagged bacteria recovered from the lymph node at a given time point, we can estimate the migration rate of bacterial cells from the gut to the cLN, and the net growth rate, 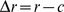, of the bacterial population in the cLN using the likelihood we derived for our model (see Materials & Methods). Unfortunately, not all three parameters of our model can be estimated independently from our data (see Materials & Methods). We still formulate the mathematical model in terms of the three processes immigration, replication, and clearance, as this is the most intuitive way to conceptualize the colonization of the cLN. Furthermore, only such a formulation allows us to understand the estimates of the process rates depend on each other (see Materials & Methods). Lastly, clearance and replication can be estimated from alternative experimental setups. Thus, a model with all three parameters may also be useful for future analysis.

### Baseline migration and replication rates

**Figure 2:**
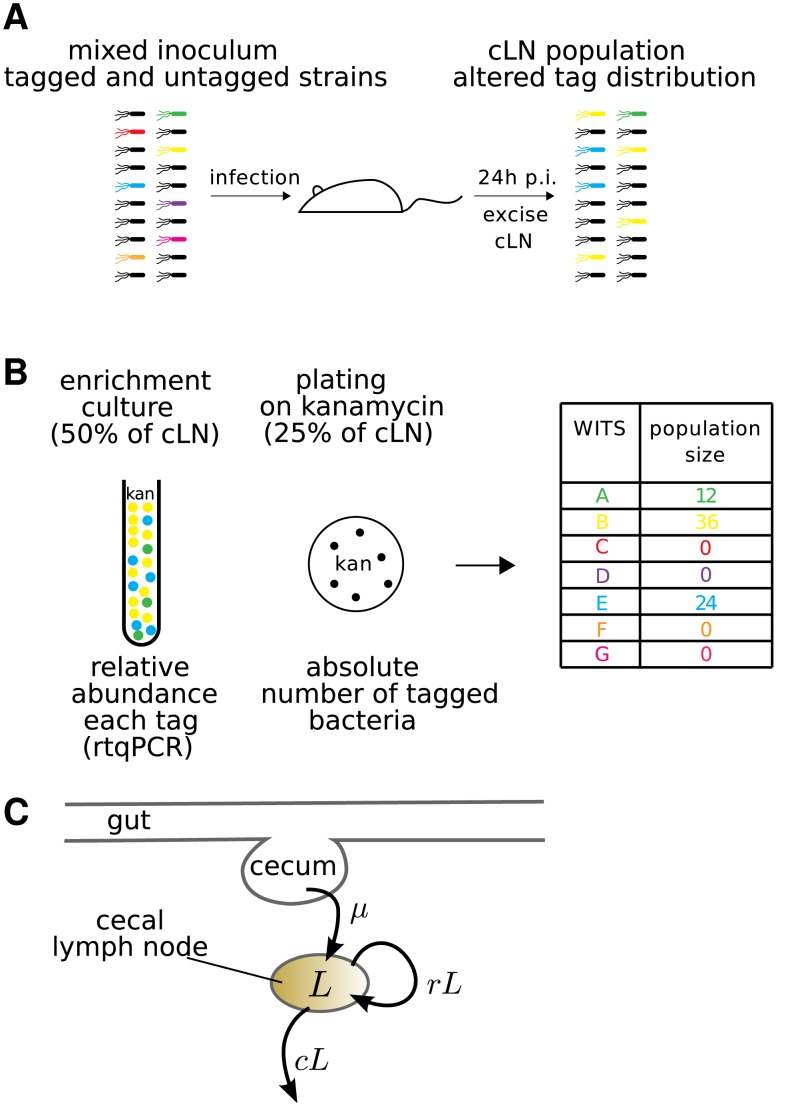
Experimental protocol for infections with mixed inocula. **A**. A mixture of all 7 WITS at equal ratios was diluted with a 20-fold excess of untagged *S.* Tm and used as an oral inoculum. The inoculum was plated to control for correct ratio of tagged to untagged bacteria. After 24 hours, the cLN was sterily removed and smashed. 50% of the cLN was inoculated into a kanamycin containing overnight culture, 25% each were plated either on a streptomycin or kanamycin containing McConkey agar plates. **B**. Typical dataset: genomic DNA of overnight culture was isolated and subjected to rtqPCR (see Materials & Methods). To obtain the population size of bacteria carrying a specific tag, the relative rtqPCR signal was multiplied with the population size of WITS in the cLN, which we determined by plating on the kanamycin plate. **C**. Diagrammatic representation of our mathematical model. The model describes the population size, 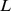, of bacteria in the cecal lymph node. It incorporates the migration of bacteria from the cecum to the cecal lymph node at a rate 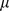, and the replication and clearance of bacteria in the lymph node at rates 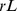 and 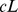, respectively. These processes are assumed to be stochastic.

We first conducted experiments in wild type C57BL/6 mice. Eleven mice were inoculated with a mixture of 95% untagged wild type *S.* Tm, and 5% WITS (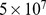 CFU in total by gavage; see Figure 2A). Each WITS carried one of seven distinct tags. These different strains were represented at equal ratios, i.e. each WITS was represented at a frequency of 0.7% in the inoculum.

Four to six hours after infection the bacteria had reached the large intestine and grew up to a population size of 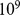. The composition of the bacterial population in the gut of the mice did not differ substantially from that of the inoculum over the course of the experiment (supporting Figure S1). Thus, the bacterial population did not encounter any detectable bottlenecks between the stomach and the gut.

**Figure 3:**
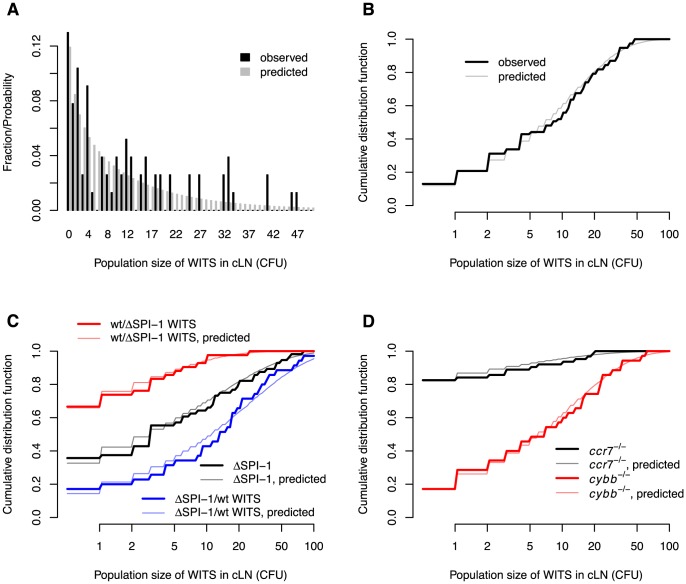
Comparison between the observed and predicted population sizes of the WITS. **A**. Comparison of observed and predicted wild type WITS population size distributions in wild type mice. **B**. The same comparison, but cumulative distribution functions of the WITS population sizes are shown. **C**. Comparison of observed and predicted WITS population size distributions in wild type mice for experiments involving 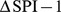. **D**. Comparison of observed and predicted WITS population size distributions in 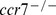 and 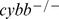 mice. The model predictions are based on the maximum likelihood estimates in Table 1.

Twenty-four hours after inoculation, the mice were sacrificed to determine the number and composition of bacteria in the cLN (see Figure 2). About 13% of the distinct WITS were lost and could not be recovered from the cLN. The population size of each WITS that successfully migrated into the cLN ranges from 1 to 47 colony forming units (CFU) (see Figure 3A).

From these numbers, we estimated that the net growth rate, 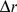, of the bacterial population is 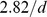, which corresponds to a doubling time of 5.9 hours (see Table 1). The migration rate of each WITS to the cLN was estimated as 2.13/d. Extrapolated to the entire bacterial population (i.e. WITS and untagged *S.* Tm), this corresponds to a migration rate of 298 cells per day. Given that the population size in the gut is approximately 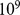 cells, this rate of migration is surprisingly low. It means that one bacterial cell has only a chance of 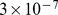 to migrate into the cLN during the first day after inoculation. This step therefore constitutes a major bottleneck for the infection.

**Table 1:** Estimates of the migration rate of bacteria into the cLN, their net replication rate, 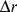, and the corresponding doubling time.

*Salmonella* strain	Mouse genotype	migration rate to cLN (per day)	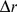 (per day)	doubling time (hours)
wild type	wild type	298 (242–391)	2.82 (2.46–3.07)	5.9 (5.4–6.8)
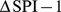	wild type	78 (50–108)	3.68 (3.15–4.09)	4.5 (4.1–5.3)
wild type, 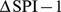 WITS (50∶1)	wild type	146 (65–242)*	2.57 (1.59–3.20)*	6.5 (5.2–10.5)*
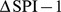 , wild type WITS (20∶1)	wild type	272 (183–412)*	3.94 (3.04–4.63)*	4.2 (3.6–5.5)*
wild type	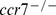	28 (11–70)	3.31 (2.96–4.19)	5.0 (4.0–5.6)
wild type	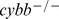	1241 (863–2050)	3.13 (2.51–3.59)	5.3 (4.6–6.6)

95% confidence intervals are given in brackets.

* values calculated for the WITS and extrapolated to the entire bacterial population.

Visualizing the agreement between the observed and predicted number of WITS in the cLN is not straight-forward. The reason for this is that our model formalizes migration and division as stochastic processes, and therefore does not make a deterministic prediction on the population size of WITS in the cLN. Rather, our model gives us the probability to observe a certain population size. For example, with the best estimates for our parameters, the predicted probability to observe 1 CFU of a single WITS in the cLN 24 hours after the infection is 0.085. The probability to observe 0 CFU, i.e. not to recover a WITS, is 0.12.

Moreover, the space of potential population sizes (any natural number) is large compared to the number of observations. In the present case, for example, we have measured the population sizes of seven WITS in 11 mice. Thus, we have 77 observations. More than half of these observations are population sizes below 10 CFU, the rest is scattered between 10 and 47. The sparseness of the observations gives rise to seeming discrepancies between the predicted and observed population sizes of WITS in the cLN 24 hours after infection (see Figure 3A).

These difficulties in visualizing the quality of the model fit can be overcome by plotting the cumulative distribution function of predicted and observed population sizes of WITS. The cumulative distribution function describes the fraction of observed or predicted measurements less than or equal to some population size. For example, we mentioned above that more than half of our observations are less than 10 CFU. This can be seen from the cumulative distribution function plotted in Figure 3B: the cumulative distribution function for a population size of 9 CFU is 0.52 and 0.58 for observed and predicted measurements of population sizes, respectively. For high population sizes, the cumulative distribution function approaches 1. Plotting these cumulative distribution functions shows the good agreement between the observed and predicted population sizes of WITS in the cLN after 24 h of infection (see Figure 3B).

To statistically test for the goodness of fit, we apply a 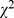 test. In order to apply this test we need define intervals of population sizes that contain a minimum of five observations per interval. For most of our datasets, population sizes 0 CFU, 1–5 CFU, 6–20 CFU, and larger than 20 CFU fulfill this criterion. For the fits of our model to the wild type WITS data, this test yields no significant discrepancy between the predicted and observed population sizes of WITS (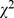 goodness-of-fit test: 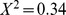, 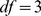, 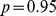).

**Figure 4:**
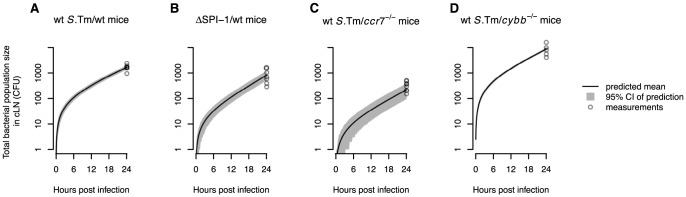
The WITS-based model predictions are consistent with the total bacterial population size measured in the cLN. Model predictions of the mean total bacterial population size in the cLN over time are shown for the different experiments. Gray areas show the 95% confidence intervals for the model predictions. **A**. Wild type mice infected with wild type *S.* Tm. **B**. Wild type mice infected with 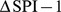. **C**. 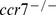 mice infected with wild type *S.* Tm. **D**. 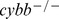 mice infected with wild type *S.* Tm. For 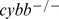 mice the confidence intervals are very narrow and therefore hardly visible. In all plots, circles denote the total bacterial population sizes observed in the cLN 24 hours after infection.

As an independent validation of our model, we can predict the total bacteria population size in the cLN (see Figure 4A). This validation is independent because the data on the total bacterial population sizes in the cLN were not taken into account when estimating the parameters. There is good agreement between the prediction and the observation: with a net growth rate of 2.82 per day and a migration rate of 298 cells per day, our model predicts a population size of, on average, 1667 bacterial cells in the cLN; in comparison, we observed a mean of 1684 cells, ranging from 950 to 2390. This concordance is quite remarkable, and provided evidence that the population parameters inferred from our WITS data analysis can be extrapolated to the entire population.

### Impact of inflammation and SPI-1

To investigate the role of inflammation, we compared the infection dynamics of wild type *S.* Tm and a non-invasive mutant *S.* Tm 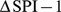 [21]. This mutant has a genetic defect rendering the type three secretion system I (TTSS-1) inactive, and hence does not cause inflammation during the course of our infection experiment [19]. We inoculated eight mice with a mix of 90% untagged, and 10% tagged strains of 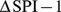. The tagged fraction contained equal proportions of seven WITS.

For the 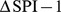 strain, we estimated a migration rate from the gut to the cLN of 78 cells per day (see Table 1). Thus the migration rate is four times lower than that of wild type *S.* Tm. The reduction of the migration rate is consistent with the key role of the SPI-1 encoded type III secretion system in traversing the gut epithelial barrier, a key step for reaching the cLN [22], [23]. Compared to wild type *S.* Tm the net replication rate of 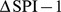 is increased from 2.82 per day to 3.68 per day. This corresponds to a decrease in the population's net doubling time from 5.9 to 4.5 hours.

The black and grey lines in Figure 3C show the cumulative distribution functions for observed and predicted WITS population sizes in the cLN. Again, there is an excellent agreement between data and model predictions (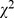 goodness-of-fit test: 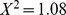, 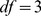, 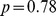). With the estimated parameters, our model can again be validated by predicting the total bacterial population size in the cLN. There is very good agreement with the experimental data, even though these measurements were not used for the parameter estimation: the model predicts an average of 819 bacterial cells in the cLN one day after inoculation, while we measured 881 cell on average, ranging from 290 to 1640 (see Figure 4B).

To investigate the cause of these differences in the immigration and net replication rates, we conducted experiments in which the inoculum consisted of wild type *S.* Tm and 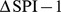. On the one hand, we used inocula, in which the large majority of the inoculum consisted of wild type *S.* Tm but the WITS lacked SPI-1. We have shown earlier that this type of infection elicits gut inflammation [24]. On the other hand, using inocula mainly consisting of 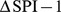 while the wild type *S.* Tm carried the tag, we could analyze the population of wild type *S.* Tm in a healthy, non-inflamed gut environment [24].

When the large majority of the inoculum consisted of wild type *S.* Tm, i.e. inflammation was induced, the population of WITS that lacked SPI-1 increased with a net doubling time of 6.5 hours (see Table 1). This estimate is similar to the net doubling time of wild type *S.* Tm (5.9 hours — see Table 1, top row), and significantly larger than the net doubling time of 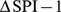 without inflammation (4.5 hours — see Table 1, second row), The migration rate estimate of 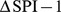 to the cLN was twice as high as without inflammation: when extrapolated to the entire population, 146 and 72 cells migrated to the cLN per day with and without inflammation, respectively. But this difference was not statistically significant.

In the converse experiment, in which the inoculum consisted mainly of 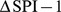 but the WITS expressed SPI-1, we estimated almost the same immigration rate as for wild type *S.* Tm (see Table 1, top row), and the same doubling time as for 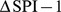 (see Table 1, second row).

The fits of the model to these two datasets are shown in Figure 3C. Both fits passed a 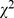 goodness of fit test (wild type, 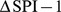 WITS: 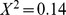, 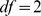, 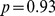; 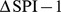, wild type WITS: 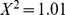, 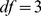, 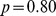).

Taken together, these experiments show that inflammation decreases the net replication rate in the cLN. The most likely explanation for this finding is an increase in bacterial clearance by immunity in the cLN. But inflammation does not significantly affect the migration between gut and cLN.

### Impact of host innate immunity

There are a number of important players in the innate immune response against *S.* Tm, including dendritic cells and phagocytes producing reactive oxygen species that are toxic to the pathogen. The general function of dendritic cells is to sample antigen, to present it to T helper cells, and thus to initiate the adaptive immune response. In *S.* Tm infections dendritic cells take up bacterial cells when they sample the gut, and subsequently carry them into the lymph node [25]–[27]. Oxygen-derived compounds are produced in phagocytes, such as neutrophils, and, to a lesser extent, in macrophages, after they have engulfed bacteria. These compounds are thought to kill the bacteria within the phagocytes, and, as a result, to reduce pathogen loads in the gut mucosa and other infected sites [24]. In summary, phagocytes have profound effects on the infection process. However, their function in controlling pathogen population dynamics had remained poorly understood.

To assess the role of dendritic cells for the early colonization dynamics with *S.* Tm we conducted infection experiments in mice lacking CCR7, in which dendritic cell movement is impaired [28]. In these 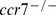 mice, we find the bacterial migration rate to the cLN to be more than 10-fold lower than in wild type animals: only 28 *S.* Tm cells migrate from the gut to the cLN per day (see Table 1). The few *S.* Tm cells that enter the cLN have a slightly, but significantly higher net replication rate than in wild type mice. As for the 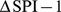, this is more likely due to reduced clearance, rather than an increased rate of division.

The fit of our model to the experimental data collected in 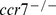 mice passes the visual and statistical test of goodness. The observed and predicted cumulative distributions of WITS population sizes coincide (see the black and gray lines in Figure 3D, respectively). The fit of our model also passed a 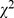 goodness of fit test (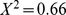, 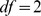, 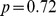). There is also good agreement between the predicted and observed the total bacterial population size in the cLN: the model predicts 220 bacterial cells, while we measured bacterial population sizes ranging from 150 to 507 cells (see Figure 4C).

The role of toxic oxygen derivatives was assessed in 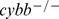 mice that have a deletion in the *p91phox* subunit of nicotinamide adenine dinucleotide phosphate oxidase, a key enzyme in the generation of reactive oxygen species. In these mice, the migration rates were four times higher than in normal mice, while there was no difference in net replication rate (see Table 1). As for all the other experiments, we observed a good agreement between model predictions and the experimental data (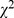 goodness-of-fit test: 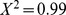, 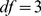, 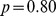; also see Figure 3D and Figure 4D).

## Discussion

In this study, we estimated dynamical parameters that characterize the colonization of the cecal lymph node of mice by *Salmonella* Typhimurium, the pathogen's first step to systemic infection. Our approach relied on infection with mixed inocula, which augmented the stochasticity of the processes involved in colonization. Exploiting this stochasticity, we could derive the rate at which the bacterial immigrate from the gut, and the net growth rate of the bacteria population in the cLN.

Performing our analysis with different bacterial strains and knock-out mice allowed us to determine the impact of various bacterial virulence factors and elements of the host's immune defense on the colonization dynamics. The inflammatory response induced by TTSS-1 was found to significantly reduce the net replication rate of bacteria in the cLN but had no significant effect on the migration rate to the cLN.

Our analysis in 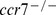 mice showed a strong and statistically significant reduction in migration to the cLN from 298 to 28 cells per day. These mice are commonly applied to assess the influence of impaired dendritic cell migration on infections and the elicitation of immune responses. Our result therefore strongly suggest that that dendritic cells play a key role in the migration of *S.* Tm from the gut to the cLN. This would be in line with previous work [25]–[27], [29], [30]. Nevertheless, since 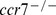 mice have a complex phenotype, alternative explanations cannot be dismissed completely.

In 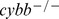 mice, which lack reactive oxygen species, migration was dramatically increased, while the net replication rate remained unchanged. The parameter estimates are in line with unaltered *S.* Tm growth within 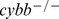 macrophages [31] and increased pathogen population sizes in the cecal lamina propria (see [24] and data not shown). These findings indicate that elimination of bacteria on their way from the gut lumen into the cLN, e.g. in the lamina propria or the transporting phagocytes, is the key factor explaining the increased migration rates to the cLN in 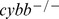 mice.

What are the limitations of our approach? Our current mathematical model is only a crude reflection of the biological realities involved in the colonization of the cecal lymph node by *S.* Tm. As any modeling study, we had to make simplifying assumptions. First, we assumed that migration rate is constant over time. This assumption is justified for migration because the population size of *S.* Tm in the gut — the source for the migration to the cLN — remains approximately constant over the period of observation. We also assumed that the replication of bacteria in the cLN occurs at a constant rate, which is a valid assumption if the population sizes do not exceed the carrying capacity of the compartment. In our case, the bacteria population in the cLN reaches about 1000 CFU within a day, which is approximately fifty fold lower than the carrying capacity of the cLN [22].

On a more subtle point, our mathematical formulation of replication and clearance implicitly assume exponentially distributed inter-division times and lifespans of bacterial cells. More realistic distributions will certainly affect the colonization dynamics. However, a reliable determination of these distributions will have to rely on individual cell measurements that were not available in this study. Extending our model by more realistic distributions of inter-division times and lifespans will be an important topic for future work.

Furthermore, our model does not explicitly account for the fact that the bacteria replicate within cells. Recent studies have started to unravel the dynamics of *Salmonella* replication within the cells of the host [32], [33]. In the future, these models will have to be combined with the mathematical description of the colonization of the host presented in this paper. Lastly, our current mathematical model neglects the detailed molecular mechanisms involved in the migration through the lamina propria.

As a consequence of the simplifying assumption we made, our model parameters have to be interpreted with care as they comprise multiple processes. The migration rate, for example, measures the effective flux of bacteria between gut and cLN. It comprises the aspects related to the movement or transport of the bacteria between these two compartments, such as bacterial penetration of the epithelial barrier, the cells they use as vehicles, their migratory speed and their sense of direction. This is important for interpreting the increase in migration rate in 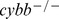 mice. Because these mice lack reactive oxygen defenses, the higher migration rate is most likely due to a decrease in killing of bacteria on their way to the cLN, and not to faster migration or transport. Similarly an increase in the net replication rate — as we found for 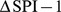 and in 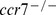 mice — can be due to faster division as well as lower rates of clearance.

At the current state of research, the main reason in favor of a simple mathematical model is to avoid over-parameterization. The higher the number of model parameters, the more difficult it is to estimate each parameter reliably. Even with three parameters, we encountered the problem that division and clearance rates were not independently identifiable. Thus, adding more complexity to the mathematical model will have to go hand in hand with experiments that provide the data necessary for reliable parameter estimation. This will be an important topic for future work.

There is a long history in microbiology of using mixed inocula to elucidate colonization dynamics of bacterial pathogens. The earliest studies on this topic [12], [13], used various strains of *Salmonella* to investigate if bacterial cells cooperated during the colonization of mice. The conclusion of these studies was that each cell had the same probability to colonize, a scenario called the *hypothesis of independent action* [34], [35]. Also our mathematical model conforms to this hypothesis. Quantitative conclusions about migration and replication were not drawn in these studies.

Later Moxon and Murphy [14] adopted the same approach for *Haemophilus* influenzae. They went beyond the first studies [12], [13] by sampling bacteria from different compartments. In addition to confirming the hypothesis of independent action, Moxon and Murphy could elucidate the colonization pathway of the pathogen. But again, as no replication and migration parameters were estimated, the conclusions of this study with regard to the colonization dynamics remained qualitative.

Margolis & Levin [16] recently investigated if bacterial evolution is required for invasive infection in the *H.* influenzae system — a hypothesis put forward in [36]. They concluded that invasion has a strong stochastic element, and the probability that a bacterial cell causes invasive infection is very low. They also find evidence for within-host evolution towards higher invasiveness. According to Margolis & Levin, the low probability of invasion and occasional evolution of invasive mutants provides an explanation of the monoclonality of invasive infections.

Our data suggest that within-host evolution towards higher invasiveness is unlikely in *S.* Tm infections. If particular mutations were necessary for the migration to the lymph node they would arise more frequently in the untagged than in the tagged population because the untagged population is at least 10 times larger in our experiments. In that case, we would be unable to extrapolate the migration rate of the WITS to that of the total bacterial population. However, the parameter estimates we obtain from the WITS allow us to predict the total bacterial population sizes in the cLN in most of our experiments (see Figure 4). Additionally, the particular migration rate we estimate (300 cells per day) implies that the systemic population of *S.* Tm is not going to be monoclonal.

More recently, WITS have been used to elucidate colonization pathways of *Yersinia* pseudotuberculosis gut infection [17] and systemic salmonellosis [18]. The main advantage of WITS over the strains used in the early studies [12]–[14] is that many distinct strains can be constructed, and that the tag is less likely to affect the phenotype, in particular the replication rate. Only in the study by Grant et al [18] estimates for parameters that characterize the within-host dynamics were obtained.

There are important differences between that previous study and our work. First, Grant et al. studied bacterial spread following intravenous infection of mice. In their model system, bacteria migrate from the blood into the spleen and the liver after inoculation. The intestine, however, is not infected efficiently and is not inflamed [22]. This system is therefore mainly of value for the study of the systemic spread of the typhoid fever-like infection. But since intravenous inoculation circumvents the epithelial barrier of the gut, the model system of Grant et al. is of limited use for the study of the first steps of the natural infection, i.e. after orogastric exposure.

Second, the estimation of the dynamical parameters in Grant et al. centered on the presence of WITS and the total bacterial population size in different anatomical compartments, and did not include information on the population size of each WITS. Their mathematical models also featured many more parameters than ours because bacterial spread and replication in multiple compartment had to be described. As a consequence, confidence intervals could not be determined for all model parameters. Without confidence intervals, differences in parameters between bacterial strains or mice genotypes cannot be established formally.

Our current approach allowed estimating key parameters of the within-host dynamics during the early phase of the diarrheal *S.* Typhimurium infection. While estimates of the dynamical parameters that characterize the colonization of the cLN are more difficult to obtain than standard readouts, such as pathogen load at some time after infection, they provide more relevant information: they tell us what happens with the bacteria over time, and thereby supply more than just snapshots of the system. Understanding the processes that the bacteria undergo is most relevant for the design of vaccines and treatment.

## Materials and Methods

### Experiments

#### Ethics statement

All animal experiments were approved by the authorities (Kantonales Veterinäramt Zürich, license: 223/2010) and performed according to local guidelines (TschV, Zürich) and the Swiss animal protection law (TschG).

#### Bacterial strains and growth conditions

The wild type strain SB300 (*S.* enterica subspecies 1 serovar Typhimurium) and the avirulent mutant 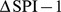 (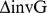) are SL1344 derivatives and have been described previously [21], [37]–[39]. Wild type isogenic tagged strains (WITS) were previously described by A.J. Grant [18]. Tags were transduced into avirulent strains using p22 phage transduction and subsequent selection on kanamycin. For infection experiments, the bacteria were grown over night in LB broth (containing 0.3 M NaCl), sub-cultured for 4 hours and suspended in cold PBS as described previously [38].

#### Wild type isogenic tagged strain quantification

Streptomycin pre-treated mice were infected according to our standard infection protocol [19] with a uniform mixture of 7 isogenic tagged strains [18]. Tagged strains were diluted with an excess of untagged *S.* Tm. The ratios of tagged to untagged strains were 1∶20 in most cases except for the experiments with 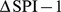, the experiments with untagged wild type *S.* Tm and 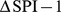 WITS, and the experiments in 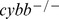 mice that were conducted with a ration of 1∶10, 1∶50, and 1∶100, respectively. Mice were killed by cervical dislocation and the cecal lymph node was aseptically removed and homogenized in 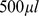 of ice-cold PBS (0.5% Tergitol, 0.5% bovine serum albumin) by using a Potter homogenizer. 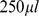 of the lymph node homogenate was inoculated into an LB overnight culture containing 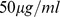 Kanamycin to enrich for tagged *Salmonella* spp and 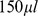 were plated on MacConkey agar plates to determine colony forming units (CFU). Chromosomal DNA from enrichment cultures was isolated using the QIAamp DNA Mini Kit (Qiagen, Cat. No. 51306) and subjected to rtqPCR using the FastStart Universal SYBR Green Master (Rox) (Roche, 13206900) mix with primers and temperature profiles described in [18]. The ratio of the WITS was multiplied with the number of CFUs recovered to determine the population size of each tagged strain in the cecal lymph node. The culturing and rtqPCR set does not bias our measure of the population size of tagged bacteria (see Figure S2).

#### Mouse experiments

Homozygous B6.129P2(C)-Ccr7tm1Rfor/J [28], homozygous B6.129S-Cybbtm1Din/J (C57BL/6 background; [40]) and wild type C57BL/6 mice were bred and kept specified pathogen free in individually ventilated cages (RCHCI, ETH Zürich). All animal experiments were approved (licences 201/2007, 223/2010, Kantonales Veterinäramt Zürich) and performed as legally required. Streptomycin pretreated mice (20 mg/animal) were infected by gavage (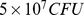; [19], [26]). Live bacterial loads (CFU) in cLN, spleen, liver, and cecal content were determined by plating [26].

### Mathematical modelling and statistical analysis

#### Model definition

To model the colonization of mice by *S.* Tm, we developed a simple stochastic model that describes the immigration of bacterial cells into the cecal lymph node (cLN) and their replication and clearance in that compartment.

The model assumes that *S.* Tm migrate from the gut to the cLN at a constant rate 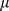. Once arrived in the cLN, a bacterial cell can divide at rate 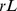 or be cleared at rate 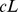. Hereby, 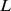 denotes the bacterial population size in the cLN. (Figure 2C shows a diagram of our mathematical model.) To keep the exposition of our model as general as possible we are, at this stage, refraining from defining which exact bacterial population the variable 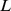 refers to. It can and will refer either to the population of WITS carrying a unique tag (when we analyze the experimental data and estimate model parameters), or to the total *S.* Tm population in the cLN (when we simulate the colonization dynamics).

To be able to describe the event that the cLN is not colonized by WITS, we formulate the model as a continuous-time Markov process. This process has the following transition probabilities:
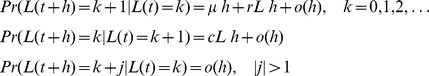
(1)Hereby, 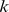 is a non-negative integer, 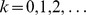, and 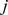 is an integer with 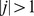. This process extends the well-known birth-death process by immigration [41].

These transition probabilities lead to the following master equations for the state probabilities, 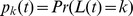:
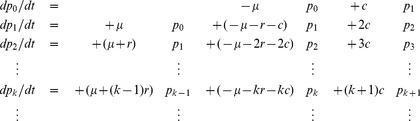
(2)Initially, we have no WITS in the cLN, which is equivalent to the initial conditions 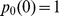 and 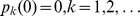.

Analogously to the birth-death process, we can derive the probability generating function that is defined as 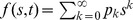. This function comprehensively characterizes the distribution of the bacterial population size in the cLN at time 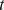. Substituting the master equations 2 into the definition of 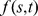 leads to a partial differential equation for 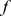 that can be solved using the method of characteristics [42]. For the initial condition 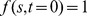, which corresponds to an initially empty lymph node (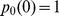 and 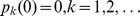), we thus obtain:
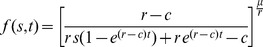
(3)


From the probability generating function we can obtain the state probabilities 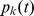 in closed form by using the relation 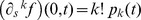. This yields:
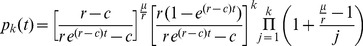
(4)
Figure S3 shows the time courses of 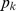 for 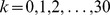. We can also obtain the mean bacterial population as a function of the time 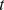, 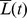, using the relation 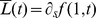:
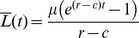
(5)


#### Maximum likelihood parameter estimation

Assume we conduct an experiment involving 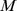 mice, each infected with an inoculum that contains a small fraction of WITS. Assume further we have seven WITS in the inoculum, and that they are present at the same frequency in the gut. Because each WITS is present at the same frequency in the gut we assume that their rate of migration into the cLN is the same. We further assume that there are no differences in the migration, replication and clearance rates between mice. We denote the universal migration rate of a WITS by 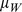.

One day after inoculation, the population sizes of WITS are determined. We denote this population size as 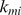, where the index 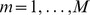 indicates the mouse in which the measure was taken, and 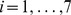 the specific WITS, the population size of which was determined. The log-likelihood for the data 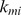 is:
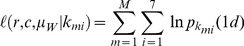
(6)

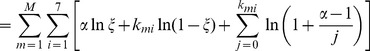
(7)Hereby 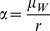 and 
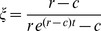
.

Maximizing this expression for the log-likelihood allows us to obtain best estimates for the parameters of our model from experimental data on the population sizes of each WITS in each mouse, 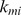. Because the expression for the log-likelihood, 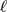, in Equation 7 depends only on the composite parameters 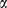 and 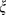, it is effectively a function of two parameters only. Thus, 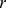, 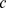, and 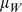 are not independently identifiable from our experimental data.

Since it is impossible to estimate all three parameters of our model, we set the clearance rate constant of bacterial cells in the cLN 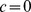, and estimated the migration rate 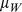 and the replication rate constant 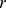. Figure S4 shows the likelihoods as functions of 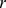 and 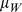 for the experiments with different *S.* Tm strains and mice genotypes. It is clear that the likelihood functions are well behaved and that there is only one maximum in the biologically relevant parameter range.

If we set 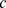 to a value larger than zero, the maximum likelihood estimate of 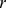 increases linearly with a slope slightly less than one (see Figure S5A). Because of this we can interpret the estimate of 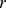 assuming 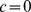 as the net growth rate, 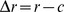. The estimate of 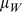 also increases with the assumed value of 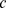, but with a slope closer to zero (see Figure S5B).

From our estimates of 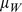 we calculate the migration rate, 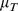, of the total bacterial population — tagged and untagged — from the gut to the cLN by dividing the estimate for a WITS by its frequency, 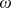: 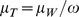. From our estimates of 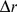 (per day) we calculate a doubling time (in hours) of the bacterial population in the cLN as 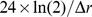. The estimates we thus obtained for 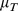, 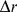, and the corresponding doubling times are given in Table 1.

#### Assessment of the goodness of fit

The quality of the fits were assessed with a 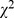 goodness of fit test [43]. To apply this test, one needs to combine population sizes into bins, in which one has more than five observation. For most of our datasets, we defined the following bins for the population sizes of WITS in the cLN: 0 CFU, 1–5 CFU, 6–20 CFU, and more than 20 CFU. We observed fewer and generally lower population sizes of WITS in the experiments involving untagged wild type *S.* Tm mixed with 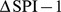 WITS and the experiments in 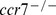 mice, To assess the goodness of fit of the data from these experiments, we therefore used the following bins: 0 CFU, 1–3 CFU, and more than 3 CFU.

The goodness of fit test compares the number of observations in the bins to the number of observations predicted by the birth-death-immigration model with the maximum likelihood parameter estimates. The null hypothesis of this test is that there is no significant discrepancy between observations and model prediction. This null hypothesis was not rejected for any of the datasets we analyzed.

#### Implementation

All the probability generating functions and likehoods described here were implemented in the R language of statistical computing [44]. To obtain the maximum likelihood estimates, we used the R-function optim(). Confidence intervals for the estimates were obtained with a bootstrap routine from 200 replicates. Goodness of fit tests were conducted using the function chisq.test() after the data and the prediction have been binned into the intervals mentioned above. To simulate the process as shown in Figure 4, we used the R-package GillespieSSA [45].

## Supporting Information

Figure S1 **Relative frequency of each WITS in the cecum 24 hours after infection.** Measurements for three representative mice are shown. All WITS strains are present at approximately equal frequencies.
(PDF)

Figure S2 **WITS at various dilutions can be detected with our protocol that involves overnight culture and rtqPCR.** There is no bias against detecting WITS at low frequencies.
(PDF)

Figure S3
**State probabilities** 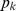 **as functions of time.** For this figure we assumed 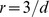, 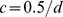, and 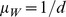.(PDF)

Figure S4 **Contour plots of likelihood for the different experiments.** The red dots indicate the maximum of the likelihood.
(PDF)

Figure S5
**Relation between assumed clearance rate constant,** 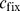 **, and our parameter estimates.** **A**. The relation to the estimate of the replication rate constant, 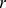 The dashed line shows the expectation under constant net replication rate 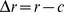. **B**. The relation to the estimate of the migration rate constant of WITS, 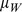.(PDF)
