# Supplementary figures and images for: Lymph Node Colonization Dynamics after Oral Salmonella Typhimurium Infection in Mice
Source: PLoS Pathog. 2013 Sep 19;9(9):e1003532. doi: 10.1371/journal.ppat.1003532 (PMC3777876; doi:10.1371/journal.ppat.1003532)

Proportions

1.0  
0.8  
0.6  
0.4  
0.2  
0.0

Mouse A

Mouse B

Mouse C

- WITS G
- WITS F
- WITS E
- WITS D
- WITS C
- WITS B
- WITS A

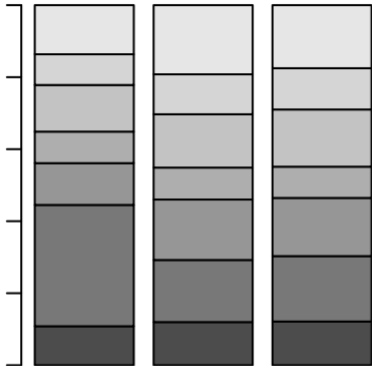

Supplement: Figure S1 — Relative frequency of each WITS in the cecum 24 hours after infection. Measurements for three representative mice are shown. All WITS strains are present at approximately equal frequencies. (PDF) [file ppat.1003532.s001.pdf]

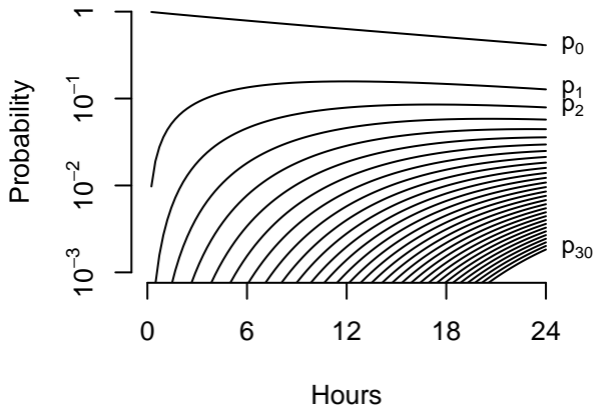

Supplement: Figure S3 — State probabilities as functions of time. For this figure we assumed , , and . (PDF) [file ppat.1003532.s003.pdf]

WITS migration rate,  $\mu_w$

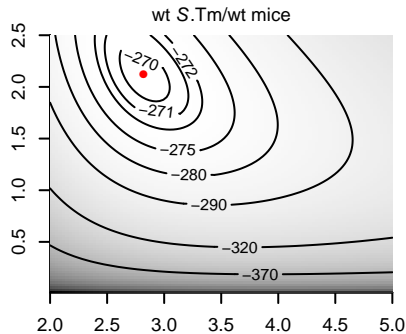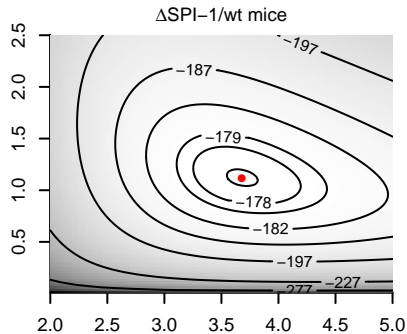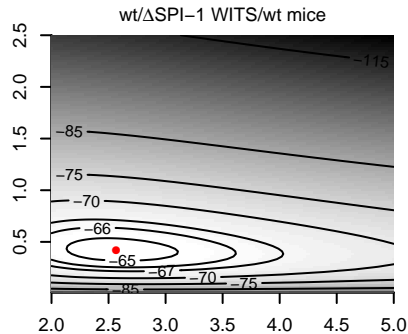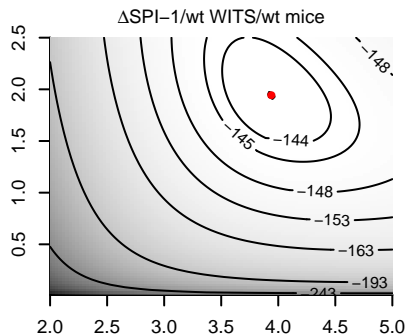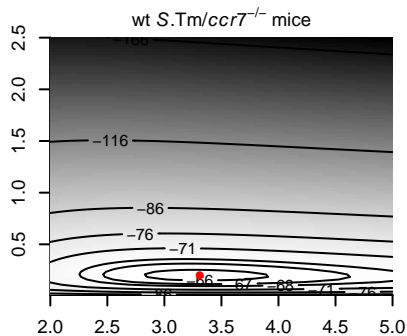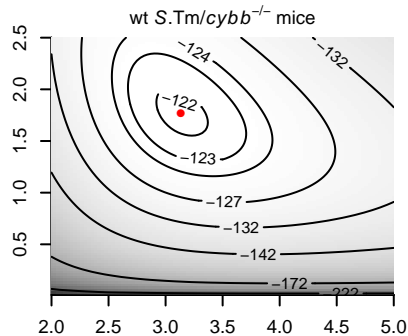

Replication rate,  $r$

Supplement: Figure S4 — Contour plots of likelihood for the different experiments. The red dots indicate the maximum of the likelihood. (PDF) [file ppat.1003532.s004.pdf]

**A**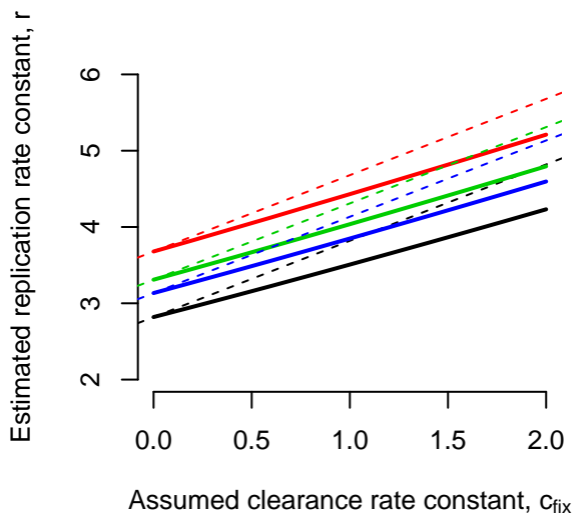**B**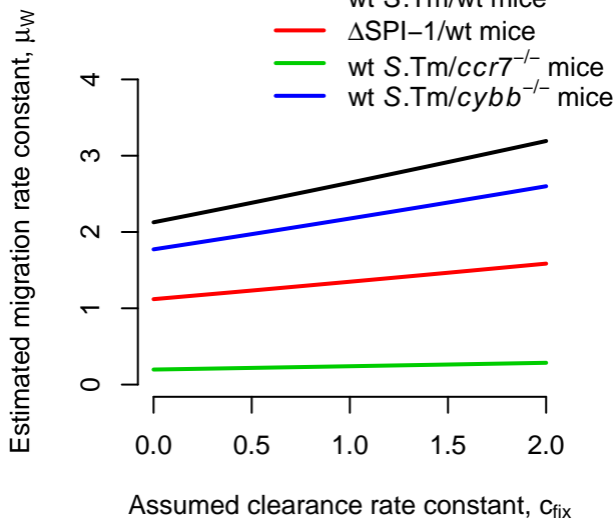

Supplement: Figure S5 — Relation between assumed clearance rate constant, , and our parameter estimates. A. The relation to the estimate of the replication rate constant, The dashed line shows the expectation under constant net replication rate . B. The relation to the estimate of the migration rate constant of WITS, . (PDF) [file ppat.1003532.s005.pdf]
