# Supplementary material for: Lymph Node Colonization Dynamics after Oral Salmonella Typhimurium Infection in Mice
Source: PLoS Pathog. 2013 Sep 19;9(9):e1003532. doi: 10.1371/journal.ppat.1003532 (PMC3777876; doi:10.1371/journal.ppat.1003532)
Supplement: Figure S2 — WITS at various dilutions can be detected with our protocol that involves overnight culture and rtqPCR. There is no bias against detecting WITS at low frequencies. (PDF) [file ppat.1003532.s002.pdf]

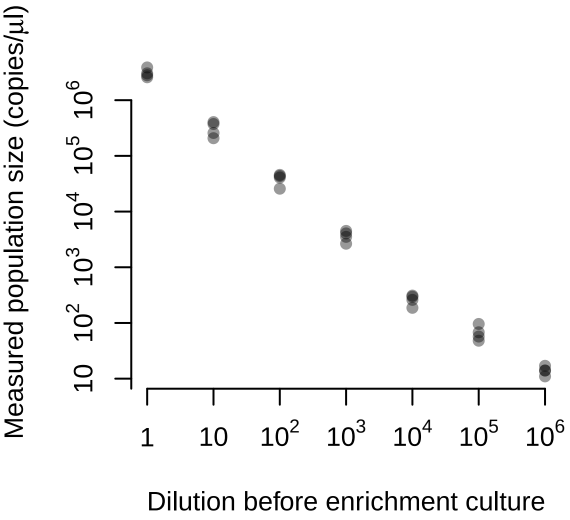
Measured population size (copies/μl)
10
10^2
10^3
10^4
10^5
10^6
1
10
10^2
10^3
10^4
10^5
10^6
Dilution before enrichment culture
